# Analysis of Mechanical Properties of Cellular Structures Under Static Tensile Loading in Standardized Specimens Manufactured by Photopolymerization

**DOI:** 10.3390/ma19142945

**Published:** 2026-07-08

**Authors:** Mateusz Rudnik, Mateusz Bronis, Mehmet Şükrü Adin, Nergizhan Anaç

**Affiliations:** 1 Department of Machine Design and Machining, Kielce University of Technology, 25314 Kielce, Poland; mbronis@tu.kielce.pl; 2 Besiri OSB Vocational School, Batman University, Batman 72060, Turkey; mehmetsukru.adin@batman.edu.tr; 3 Faculty of Engineering, Zonguldak Bülent Ecevit University, Zonguldak 67100, Turkey; nergizhan.kavak@beun.edu.tr

**Keywords:** cellular structures, photopolymerization, static tensile test, mechanical properties, *PolyJet Matrix*, additive manufacturing

## Abstract

This study investigates the mechanical behavior and anisotropy of cellular structures fabricated using *PolyJet Matrix* (*PJM*) technology from *RGD 720* photopolymer resin. Standard *ISO 527* specimens were produced at build orientations of 0°, 45°, and 90° to evaluate the influence of printing direction on tensile properties. Based on these results, the optimal 0° orientation was selected for further analysis of cellular structures, including hexagonal, spiral, and quasi-self-similar geometries, manufactured in both unfilled and silicone-filled configurations. Static tensile tests were performed to determine load–displacement characteristics, maximum load, and deformation behavior. The results reveal a strong dependence of mechanical properties on build orientation, with the highest strength observed at 0° and the lowest at 90°, confirming significant material anisotropy. This behavior was further quantified using first- and second-order anisotropy coefficients derived from experimental data. The introduction of silicone filling improved load-bearing capacity, reduced variability, and promoted a more ductile failure mechanism. Among the analyzed geometries, quasi-self-similar structures exhibited the best mechanical performance, while unfilled structures showed lower strength and higher deformation. The findings demonstrate that both build orientation and structural design are critical factors in optimizing the mechanical properties of additively manufactured components and provide a basis for designing tailored cellular structures for engineering applications.

## 1. Introduction

Additive manufacturing (*AM*) has become a key technology in modern engineering, enabling the fabrication of complex geometries with reduced material waste and high design flexibility [1]. Among various *AM* techniques, material extrusion methods such as *Fused Deposition Modeling* (*FDM*) and *Continuous Filament Fabrication* (*CFF*) are widely used due to their accessibility and versatility in processing thermoplastic materials such as *PLA*, *ABS*, and *PETG* [2,3]. However, the layer-by-layer fabrication principle inherently introduces anisotropy, leading to mechanical properties that strongly depend on printing parameters and build orientation [2,4].

A significant body of research has focused on optimizing process parameters to improve the mechanical performance of additively manufactured parts. Parameters such as infill density, layer thickness, raster angle, printing temperature, and speed have been identified as critical factors affecting tensile strength, stiffness, and ductility [4,5,6,7]. In particular, infill density and printing orientation are often reported as dominant variables, with higher infill and favorable orientations (e.g., flat or on-edge) resulting in improved strength, while upright orientations typically lead to reduced mechanical performance due to weaker interlayer bonding [2,4]. Additionally, microstructural defects such as voids between deposited filaments and imperfect interlayer adhesion have been shown to significantly influence failure mechanisms and reduce strength [8].

Beyond standard parameter optimization, several studies have explored advanced approaches to enhance mechanical properties. These include reinforcement with continuous fibers in *CFF* technology, which can significantly increase strength but introduces limitations in thin-walled structures [9], as well as process modifications such as induced vibrations to reduce porosity and improve bonding [3]. Furthermore, statistical and analytical methods, including *ANOVA* and design of experiments (*DOE*), have been applied to quantify the influence of process variables and identify optimal parameter combinations [4,5,10].

Despite extensive research on *FDM*-based materials, most studies focus primarily on process parameter optimization rather than structural design or material hybridization. Moreover, the majority of investigations address solid or infill-based geometries, while relatively limited attention has been given to cellular structures and multi-material systems, particularly in technologies other than *FDM*. In addition, while anisotropy is well-recognized, its origin is typically attributed to interlayer bonding rather than the combined effect of material architecture and structural topology [11,12,13].

Recent studies have demonstrated that PolyJet Matrix (PJM) technology offers unique capabilities for manufacturing high-resolution components with excellent dimensional accuracy, superior surface quality, and the possibility of simultaneously processing multiple photopolymer materials within a single manufacturing process. These features have enabled the development of advanced multi-material systems, voxel-based composite architectures, and complex cellular structures with tailored mechanical performance. In addition to engineering applications, PolyJet technology has also been successfully adopted in biomedical engineering, where high geometric fidelity and material customization are required. Recent investigations have further focused on the mechanical behavior, rheological and viscoelastic properties of cellular structures manufactured using PJM technology, demonstrating the significant influence of material distribution and structural architecture on the overall mechanical response. Despite these advances, studies simultaneously addressing the combined influence of build orientation, cellular geometry, and hybrid material systems on the tensile behavior and anisotropy of PolyJet-manufactured structures remain limited [14,15,16,17,18].

In contrast to *FDM*-based approaches, *PolyJet Matrix* (*PJM*) technology enables the fabrication of multi-material and complex cellular structures with high resolution, offering new possibilities for tailoring mechanical properties through structural design rather than solely through process parameters. Compared to conventional material extrusion techniques such as *FDM*, *PolyJet Matrix* technology offers significantly higher printing resolution, improved surface quality, and the capability of manufacturing complex multi-material structures with thin-walled architectures. These advantages make *PJM* particularly attractive for engineering applications requiring high geometric accuracy and tailored mechanical response. Furthermore, the ability to combine rigid and compliant materials within a single manufacturing process enables the fabrication of hybrid cellular systems with enhanced functional performance. Nevertheless, despite these advantages, the layer-by-layer deposition mechanism in PJM still introduces anisotropic behavior and interfacial effects that may significantly influence mechanical performance [19,20].

However, the mechanical behavior and anisotropy of such hybrid structures, especially under tensile loading, remain insufficiently explored. To the best of the authors’ knowledge, limited studies have investigated the combined influence of build orientation, cellular geometry, and silicone filling on the tensile behavior of *PolyJet Matrix*-manufactured *RGD 720* structures. Furthermore, existing studies rarely consider anisotropy assessment methods that incorporate both force-related and deformation-related parameters simultaneously.

The investigated structures represent thin-walled cellular architectures characterized by complex deformation and progressive failure mechanisms. Therefore, global load–displacement behavior was considered particularly suitable for evaluating structural performance, deformation stability, and energy absorption capability.

Therefore, the aim of this study is to investigate the tensile behavior and anisotropy of thin-walled cellular structures manufactured using *PJM* technology. Unlike previous works that rely mainly on conventional statistical optimization methods such as *ANOVA*, the proposed methodology is based not only on conventional anisotropy coefficients associated with force parameters, but also on matrix formulations incorporating both force and displacement values to systematically analyze the influence of structural configuration and material composition, including the effect of silicone-filled cellular architectures. This approach enables a more comprehensive understanding of the relationship between structure, material, deformation behavior, and mechanical response in additively manufactured cellular systems.

## 2. Materials and Methods

### 2.1. PolyJet Matrix Technology

In the case of the *Connex350* (Stratasys Ltd., Rehovot, Israel) 3D printer, most process parameters are predefined and automatically set by the manufacturer. Each type of processed polymer has a predetermined layer thickness that can be deposited, which results from the complex physics of droplet formation in photopolymer jetting. The typical layer thickness of the deposited material ranges from 16 to 30 µm. It is also worth noting that the manufacturer provides three operating modes for the 3D printer (*High Speed*, high build rate with a layer thickness of 30 µm; *High Quality*, lower printing speed but very high accuracy due to a layer thickness of 16 µm; *Digital Material*: enabling the combination of different materials at a layer thickness of 30 µm) [21,22].

### 2.2. Materials

The conducted research is based on the *ISO 527* standard [23]. These tests enable the assessment of mechanical properties and support the optimization of 3D printing processes to obtain structures with desired functional characteristics. Within the scope of the study, *RGD 720* (photopolymer resin) and molding silicone (elastomeric material) were used. Each material exhibits distinct properties, as presented below (Table 1), based on the manufacturer’s datasheets.

### 2.3. Mathematical Formulation

The arithmetic mean and standard deviation formulas were used to determine the relevant characteristics:

(1) $$\bar{X}=\frac{1}{n}\sum_{i=1}^{n}x_{i}\;$$

(2) $$SD=\sqrt{\frac{1}{n-1}\sum_{i=1}^{n}{\left(x_{i}-\bar{x}\right)}^{2}}$$

where $\bar{X}$—mean value, *n*—number of samples, and *x_i_*—individual data values.

For each specimen, the cross-sectional area (3) was calculated in accordance with Equation (3):

*S*_0_ = *b*_1_·*h* (3)

where *S*_0_—cross-sectional area of the *ISO 527* specimen (mm^2^), *b*_1_—specimen thickness (mm), *h*—specimen height (mm).

For the purposes of this study, first- and second-order anisotropy coefficients were determined. The first-order anisotropy coefficients were introduced to evaluate directional differences in load–bearing capacity, whereas the second-order coefficients additionally incorporated displacement-related deformation behavior characteristic of thin-walled cellular structures. These coefficients take into account the mean maximum load values, and in the case of the second-order anisotropy coefficient, as well as the displacement.

To determine the first-order anisotropy coefficient (*A_I_*) and to characterize the material based on the matrix determinant, original formulations were applied, defined and adopted specifically for this study (4)–(11):

(4) $${A_{I}}_{0{}^{\circ}/45{}^{\circ}}^{RGD\;720}=\;\frac{\bar{F_{m0^{{}^{\circ}}}}}{\bar{F_{m45^{{}^{\circ}}}}}$$

(5) $${A_{I}}_{0{}^{\circ}/90{}^{\circ}}^{RGD\;720}=\frac{\bar{F_{m0^{{}^{\circ}}}}}{\bar{\bar{F_{m90^{{}^{\circ}}}}}}$$

(6) $${A_{I}}_{45{}^{\circ}/90{}^{\circ}}^{RGD\;720}=\frac{\bar{F_{m45^{{}^{\circ}}}}}{\bar{F_{m90^{{}^{\circ}}}}}$$

(7) $${A_{I}}_{45{}^{\circ}/0{}^{\circ}}^{RGD\;720}=\frac{\bar{F_{m45^{{}^{\circ}}}}}{\bar{F_{m0{}^{\circ}}}}$$

(8) $${A_{I}}_{90{}^{\circ}/0{}^{\circ}}^{RGD\;720}=\frac{\bar{F_{m90^{{}^{\circ}}}}}{\bar{F_{m0^{{}^{\circ}}}}}$$

(9) $${A_{I}}_{90{}^{\circ}/45{}^{\circ}}^{RGD\;720}=\frac{\bar{F_{{m90}^{{}^{\circ}}}}}{\bar{F_{m45^{{}^{\circ}}}}}$$

(10) $$M_{A_{I}}=\left(\begin{array}{ccc} \begin{array}{c} {A_{I}}_{0^{{}^{\circ}}/0^{{}^{\circ}}}^{RGD\;720}\\ {A_{I}}_{0^{{}^{\circ}}/1^{{}^{\circ}}}^{RGD\;720}\\ \begin{array}{c} \vdots \\ {A_{I}}_{0^{{}^{\circ}}/90^{{}^{\circ}}}^{RGD\;720} \end{array} \end{array} & \begin{array}{c} {A_{I}}_{1^{{}^{\circ}}/0^{{}^{\circ}}}^{RGD\;720}\\ {A_{I}}_{1^{{}^{\circ}}/1^{{}^{\circ}}}^{RGD\;720}\\ \begin{array}{c} \vdots \\ {A_{I}}_{1^{{}^{\circ}}/90^{{}^{\circ}}}^{RGD\;720} \end{array} \end{array} & \begin{array}{cc} \begin{array}{c} \ldots \\ \ldots \\ \begin{array}{c} \ddots \\ \ldots \end{array} \end{array} & \begin{array}{c} {A_{I}}_{90^{{}^{\circ}}/0^{{}^{\circ}}}^{RGD\;720}\\ {A_{I}}_{90^{{}^{\circ}}/1^{{}^{\circ}}}^{RGD\;720}\\ \begin{array}{c} \vdots \\ {A_{I}}_{90^{{}^{\circ}}/90^{{}^{\circ}}}^{RGD\;720} \end{array} \end{array} \end{array} \end{array}\right)$$

(11) $$\left|W_{A_{I}}\right|=\left|\begin{array}{ccc} \begin{array}{c} {A_{I}}_{0^{{}^{\circ}}/0^{{}^{\circ}}}^{RGD\;720}\\ {A_{I}}_{0^{{}^{\circ}}/1^{{}^{\circ}}}^{RGD\;720}\\ \begin{array}{c} \vdots \\ {A_{I}}_{0^{{}^{\circ}}/90^{{}^{\circ}}}^{RGD\;720} \end{array} \end{array} & \begin{array}{c} {A_{I}}_{1^{{}^{\circ}}/0^{{}^{\circ}}}^{RGD\;720}\\ {A_{I}}_{1^{{}^{\circ}}/1^{{}^{\circ}}}^{RGD\;720}\\ \begin{array}{c} \vdots \\ {A_{I}}_{1^{{}^{\circ}}/90^{{}^{\circ}}}^{RGD\;720} \end{array} \end{array} & \begin{array}{cc} \begin{array}{c} \ldots \\ \ldots \\ \begin{array}{c} \ddots \\ \ldots \end{array} \end{array} & \begin{array}{c} {A_{I}}_{90^{{}^{\circ}}/0^{{}^{\circ}}}^{RGD\;720}\\ {A_{I}}_{90^{{}^{\circ}}/1^{{}^{\circ}}}^{RGD\;720}\\ \begin{array}{c} \vdots \\ {A_{I}}_{90^{{}^{\circ}}/90^{{}^{\circ}}}^{RGD\;720} \end{array} \end{array} \end{array} \end{array}\right|$$

where $\bar{F_{mi}}$—mean maximum load during the tensile test, *φ*—build orientation angle (°), $M_{A_{I}}$—first-order anisotropy matrix, and $\left|W_{A_{I}}\right|$—determinant of the first-order anisotropy matrix.

Equations (4)–(11) define the first-order anisotropy coefficients based on the ratios of the mean maximum loads obtained for different build orientations (0°, 45°, and 90°). The matrix $M_{AI}$ represents the first-order anisotropy matrix, whereas $W_{AI}$ denotes the determinant of the first-order anisotropy matrix. In the matrix, the first element corresponds to the 0°/0° configuration, while the numerator orientation changes across the rows and the denominator orientation changes across the columns.

To determine the second-order anisotropy coefficient (*A_II_*) and to characterize the material based on the matrix determinant, Equations (12)–(19) were applied:

(12) $${A_{II}}_{0{}^{\circ}/45{}^{\circ}}^{RGD\;720}=\;\frac{\bar{F_{m0}}{}^{\circ}\cdot \bar{{∆L}_{0{}^{\circ}}}}{\bar{F_{m45{}^{\circ}}}\cdot \bar{{∆L}_{45{}^{\circ}}}}$$

(13) $${A_{II}}_{0{}^{\circ}/90{}^{\circ}}^{RGD\;720}=\frac{\bar{F_{m0}{}^{\circ}}\cdot \bar{{∆L}_{0{}^{\circ}}}}{\bar{F_{m90{}^{\circ}}}\cdot \bar{∆L_{90{}^{\circ}}}}$$

(14) $${A_{II}}_{45{}^{\circ}/90{}^{\circ}}^{RGD\;720}=\frac{\bar{F_{m45}{}^{\circ}}\cdot \bar{{∆L}_{45{}^{\circ}}}}{\bar{F_{m90{}^{\circ}}}\cdot \bar{{∆L}_{90{}^{\circ}}}}$$

(15) $${A_{II}}_{45{}^{\circ}/0{}^{\circ}}^{RGD\;720}=\frac{\bar{F_{m45}}{}^{\circ}\cdot \bar{{∆L}_{45{}^{\circ}}}}{\bar{F_{m0{}^{\circ}}}\cdot \bar{{∆L}_{0{}^{\circ}}}}$$

(16) $${A_{II}}_{90{}^{\circ}/0{}^{\circ}}^{RGD\;720}=\frac{\bar{F_{m90}{}^{\circ}}\cdot \bar{{∆L}_{90{}^{\circ}}}}{\bar{F_{m0{}^{\circ}}}\cdot \bar{∆L_{0{}^{\circ}}}}$$

(17) $${A_{II}}_{90{}^{\circ}/45{}^{\circ}}^{RGD\;720}=\frac{\bar{F_{m90}{}^{\circ}}\cdot \bar{{∆L}_{90{}^{\circ}}}}{\bar{F_{m45{}^{\circ}}}\cdot \bar{{∆L}_{45{}^{\circ}}}}$$

(18) $$M_{A_{II}}=\left(\begin{array}{ccc} \begin{array}{c} {A_{II}}_{0^{{}^{\circ}}/0^{{}^{\circ}}}^{RGD\;720}\\ {A_{II}}_{0^{{}^{\circ}}/1^{{}^{\circ}}}^{RGD\;720}\\ \begin{array}{c} \vdots \\ {A_{II}}_{0^{{}^{\circ}}/90^{{}^{\circ}}}^{RGD\;720} \end{array} \end{array} & \begin{array}{c} {A_{II}}_{1^{{}^{\circ}}/0^{{}^{\circ}}}^{RGD\;720}\\ {A_{II}}_{1^{{}^{\circ}}/1^{{}^{\circ}}}^{RGD\;720}\\ \begin{array}{c} \vdots \\ {A_{II}}_{1^{{}^{\circ}}/90^{{}^{\circ}}}^{RGD\;720} \end{array} \end{array} & \begin{array}{cc} \begin{array}{c} \ldots \\ \ldots \\ \begin{array}{c} \ddots \\ \ldots \end{array} \end{array} & \begin{array}{c} {A_{II}}_{90^{{}^{\circ}}/0^{{}^{\circ}}}^{RGD\;720}\\ {A_{II}}_{90^{{}^{\circ}}/1^{{}^{\circ}}}^{RGD\;720}\\ \begin{array}{c} \vdots \\ {A_{II}}_{90^{{}^{\circ}}/90^{{}^{\circ}}}^{RGD\;720} \end{array} \end{array} \end{array} \end{array}\right)$$

(19) $$\left|W_{A_{II}}\right|=\left|\begin{array}{ccc} \begin{array}{c} {A_{II}}_{0^{{}^{\circ}}/0^{{}^{\circ}}}^{RGD\;720}\\ {A_{II}}_{0^{{}^{\circ}}/1^{{}^{\circ}}}^{RGD\;720}\\ \begin{array}{c} \vdots \\ {A_{II}}_{0^{{}^{\circ}}/90^{{}^{\circ}}}^{RGD\;720} \end{array} \end{array} & \begin{array}{c} {A_{II}}_{1^{{}^{\circ}}/0^{{}^{\circ}}}^{RGD\;720}\\ {A_{II}}_{1^{{}^{\circ}}/1^{{}^{\circ}}}^{RGD\;720}\\ \begin{array}{c} \vdots \\ {A_{II}}_{1^{{}^{\circ}}/90^{{}^{\circ}}}^{RGD\;720} \end{array} \end{array} & \begin{array}{cc} \begin{array}{c} \ldots \\ \ldots \\ \begin{array}{c} \ddots \\ \ldots \end{array} \end{array} & \begin{array}{c} {A_{II}}_{90^{{}^{\circ}}/0^{{}^{\circ}}}^{RGD\;720}\\ {A_{II}}_{90^{{}^{\circ}}/1^{{}^{\circ}}}^{RGD\;720}\\ \begin{array}{c} \vdots \\ {A_{II}}_{90^{{}^{\circ}}/90^{{}^{\circ}}}^{RGD\;720} \end{array} \end{array} \end{array} \end{array}\right|$$

where $\bar{F_{mi}}$—mean maximum load during the tensile test (N), $\bar{∆L}$—mean maximum displacement at specimen failure recorded during the tensile test (mm), *φ*—build orientation angle (°), $M_{A_{II}}$—second-order anisotropy matrix, and $\left|W_{A_{II}}\right|$—determinant of the second-order anisotropy matrix.

Equations (12)–(17) define the second-order anisotropy coefficients as ratios of the products of the mean maximum load and mean displacement values corresponding to individual build orientations. Analogously to the first-order anisotropy formulation, the second-order anisotropy matrix $M_{AII}$ and its determinant $W_{AII}$ were introduced. The proposed anisotropy coefficients were introduced to quantify differences in the mechanical response of specimens manufactured at different build orientations. The first-order anisotropy coefficients describe the relative changes in maximum tensile force between individual orientations, whereas the second-order coefficients additionally incorporate displacement-related deformation behavior, thereby accounting for the energy absorbed during loading. The anisotropy matrices $M_{AI}$ and $M_{AII}$ were formulated to provide a compact mathematical representation of the global directional mechanical response of the investigated structures. Each row and column corresponds to a specific build orientation, while each matrix element represents a normalized relationship between the mechanical responses obtained for two different orientations. Consequently, the complete matrix describes the mutual interactions among all analyzed printing directions rather than isolated pairwise comparisons, enabling a comprehensive assessment of directional mechanical behavior.

The determinant of the anisotropy matrix was adopted as a global descriptor of anisotropy because, from the viewpoint of linear algebra, it reflects the degree of linear independence of the matrix rows (or columns), which represent the directional mechanical responses. Determinant magnitudes approaching zero indicate that the responses corresponding to different build orientations become nearly linearly dependent, suggesting relatively weak anisotropy and similar mechanical behavior irrespective of printing direction. Conversely, increasing determinant magnitudes indicate progressively greater differentiation between directional responses and, therefore, a higher degree of anisotropy.

The sign of the determinant is not assigned a direct physical interpretation in the present study because it depends solely on the ordering of the matrix rows and columns. Consequently, the assessment of anisotropy is based on the determinant magnitude rather than its sign.

The proposed anisotropy assessment methodology should be interpreted as a comparative engineering approach intended to evaluate directional differences in mechanical response under static tensile loading conditions. The introduced anisotropy coefficients and matrix determinants do not represent constitutive material parameters; instead, they provide comparative descriptors of the force- and energy-related responses associated with different build orientations.

### 2.4. Samples Preparation

**Figure 1:**
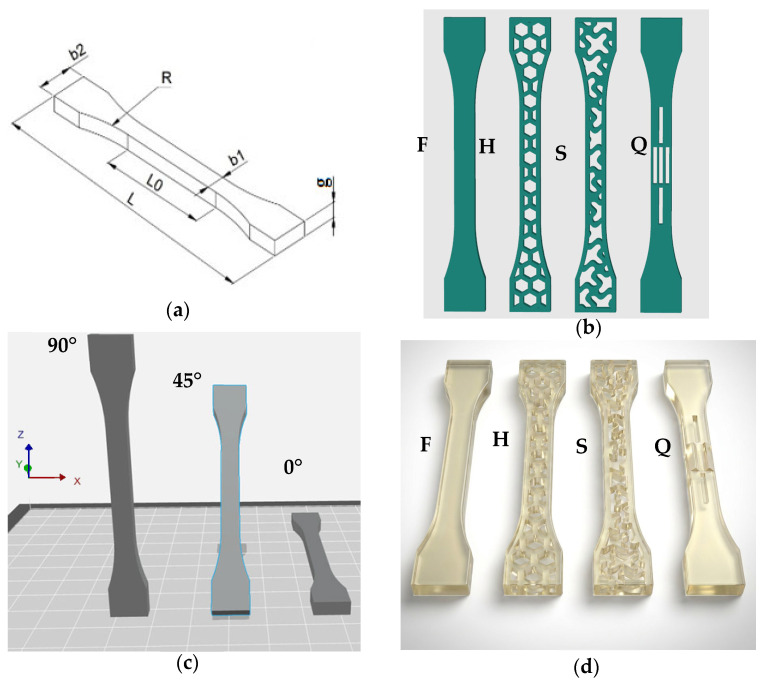
*ISO 527* specimen: (**a**) specimen dimensions; (**b**) specimen types: solid (F), hexagonal (H), spiral (S), quasi-self-similar (Q); (**c**) build orientation on the platform: 90°, 45°, 0°; (**d**) examples of four specimens manufactured using *PolyJet Matrix* (*PJM*) technology.

The static tensile test is one of the fundamental methods for evaluating the mechanical properties of materials. These tests are based on established equations that enable the determination of tensile strength, Young’s modulus, and other mechanical parameters. In this study, the *ISO 527* standard was applied, which defines the specimen geometry. According to the designations (Figure 1a), the specimen dimensions were as follows: L = 75 mm, L_0_ = 30 mm, R = 40.45 mm, b_1_ = 5 mm, b_2_ = 10 mm, and g = 4 mm. Prior to each tensile test, the geometric parameters b_1_ and g were measured. Three types of cellular structures, designated H, S and Q, were selected.

The adopted geometric parameters of the investigated cellular structures resulted from the assumptions of the broader doctoral research framework, which included analyses of single-cell geometries, as well as compression, tensile, compression relaxation, and bending tests. The selected configurations enabled comparative evaluation of structural efficiency and mechanical performance while reducing the overall specimen mass.

For tensile testing in accordance with *ISO 527*, three types of *RGD 720* specimens were prepared, printed at build orientations of 0°, 45°, and 90°, as well as six types of specimens containing cellular structures (hexagonal, spiral, and quasi-self-similar), in two variants: base material and base material with silicone filling (Figure 1b,c).

The spiral structure presented in Figure 1b corresponds to the actual geometry used in the experimental study and not only to a schematic representation. The asymmetrical arrangement of the structure along the specimen length resulted from the adopted design assumptions and was intentionally selected to reduce material consumption while maintaining the desired structural characteristics. The quasi-self-similar structure was inspired by the concept of fractal self-similarity, where smaller regions resemble selected features of the overall geometry at a different scale. However, the proposed geometry represents an engineering approximation adapted for additive manufacturing and mechanical testing conditions rather than a mathematically ideal fractal structure.

The distribution of the quasi-self-similar (Q) cellular structure was intentionally limited to the gauge section of the specimen. This design was based on the concept of geometric self-similarity derived from fractal geometry, in which the rectangular voids were designed to be geometrically similar to the rectangular gauge section subjected to tensile loading. Consequently, the cellular architecture was intentionally confined to the mechanically active region of the specimen. In contrast, the H and S topologies were generated according to different geometric principles, allowing their characteristic cellular patterns to extend into the shoulder regions while preserving their structural continuity. The objective of the present study was to compare representative cellular topologies rather than structures with identical relative density or cellular volume fraction. Therefore, each topology retained its characteristic geometric features while the external specimen dimensions and manufacturing conditions remained identical.

Figure 1c presents the build orientations used during specimen manufacturing. The printing process was performed in the *XYZ* coordinate system of the *PolyJet Matrix* printer, where the layer deposition direction corresponded to the Z-axis, while the build platform movement occurred along the Y-axis. The tensile loading direction during the static tensile test was defined along the longitudinal axis of the specimen. Consequently, the relative orientation between deposited layers and the tensile loading direction differed for the 0°, 45°, and 90° configurations. Three types of cellular structures, denoted by H, S, and Q, were selected. These structures were integrated into standardized specimens to evaluate their influence on mechanical properties. The specimens were fabricated using *PolyJet Matrix* technology on a *Connex350* (Stratasys Ltd., Rehovot, Israel) printer in *High Quality* (*HQ*) mode, ensuring high dimensional accuracy and surface quality. The layer thickness was 16 µm, enabling precise reproduction of structural features and minimizing surface defects.

The conducted study provides significant data on the mechanical properties of selected cellular structures as a function of build orientation and the type of cellular architecture applied. These results can be utilized for the optimization of additive manufacturing processes and for the design of materials with tailored mechanical properties, which is of broad relevance across various industrial sectors.

The specimens were fabricated using *RGD 720*, a photopolymer resin, and subsequently subjected to static tensile testing on an *Inspekt Mini 3 kN* testing machine (Hegewald & Peschke MPT GmbH, Nossen, Germany). An additional aspect addressed in this research was the determination of the anisotropy coefficient, which quantifies differences in mechanical properties depending on the loading direction, as well as the development of a mathematical model for the *RGD 720* material.

For the silicone-filled configurations, a dedicated mold reproducing the geometry of the specimen was manufactured using additive technology. The molding silicone was mixed with a catalyst and poured into the mold, after which the printed cellular structure was inserted into the silicone-filled cavity. The curing process was conducted at room temperature (22 °C) for approximately 10 min. No additional air bubble removal procedure was required, as no visible air inclusions were observed during specimen preparation. The applied procedure ensured good repeatability of the filling process.

### 2.5. Inspekt Mini 3kN

The single-column universal testing machine *Inspekt Mini 3 kN*, equipped with *LabMaster* software 2.7.4.8 version, was used for measurements in the range of low to medium loads. The device utilizes an AC motor drive and enables testing up to a maximum load of 3 kN. A working space depth of 105 mm and a maximum crosshead displacement of 850 mm (excluding grips and load cell) provide flexibility in configuring the test setup. The position measurement resolution of 0.001 mm allows for precise recording of specimen deformation [26].

## 3. Results

### 3.1. Static Tensile Behavior of RGD 720 Specimens

**Figure 2:**
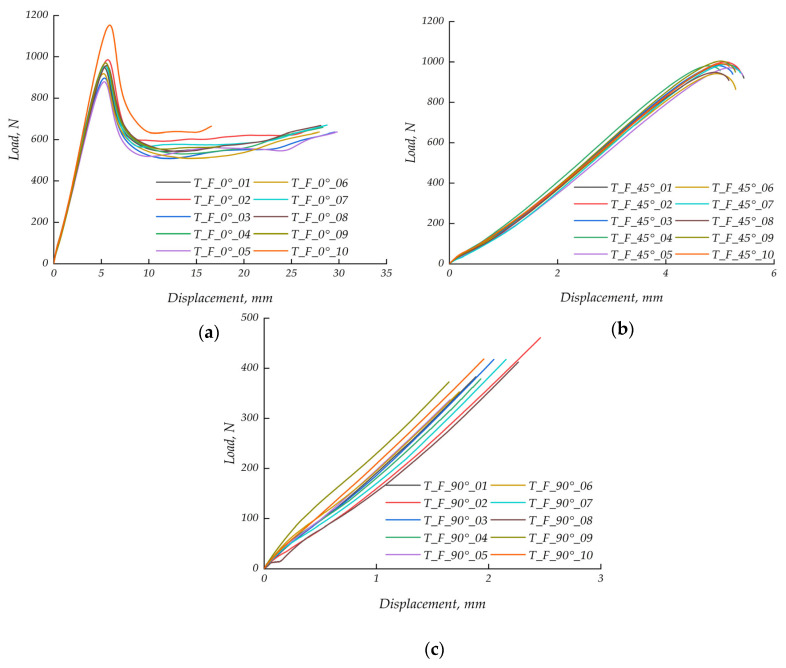
Load–displacement curves from the static tensile test of *RGD 720* specimens with build orientations: (**a**) 0°; (**b**) 45°; (**c**) 90°.

Within the experiment, three types of specimens printed at three different build orientations (0°, 45°, and 90°) were investigated. For each configuration, 10 specimens were tested. Each specimen type was analyzed to evaluate the influence of build orientation on the mechanical properties of the material. Subsequently, using a selection approach, specimens printed at 0° containing cellular structures were chosen for further analysis. *ISO 527* specimens with a length of L = 75 mm were manufactured in the 0° build orientation. All tested specimens (*n* = 10) exhibited a rapid increase in load within the first few millimeters of displacement, reaching a maximum in the range of approximately 900–1200 N. The peak load occurred at displacements between approximately 2 and 6 mm, followed by a decrease in load. After reaching the maximum value, the load decreased gradually and stabilized at approximately 600–700 N for further displacements up to 30 mm. The results for individual specimens are relatively consistent, although slight variations in maximum load and post-peak behavior are observed (Figure 2a). All recorded curves corresponding to *n* = 10 specimens for each configuration are presented in Figure 2. Due to the high repeatability of the experimental results, several curves partially overlap.

The plot shown in Figure 2b presents the results of static tensile tests for ten specimens (*n* = 10) printed from *RGD 720* photopolymer resin at a 45° build orientation. All specimens exhibit similar mechanical behavior. The load increases rapidly at the initial stage of the test, corresponding to elastic deformation. As displacement increases, the load rises more gradually, reaching maximum values in the range of 900–1100 N at displacements of approximately 4–5 mm. After reaching the peak, a sharp drop in load is observed, indicating material failure. Compared with the 0° build orientation, the specimens manufactured at 45° exhibited lower displacement at failure. This behavior may be attributed to the combined action of normal and shear stresses acting at the interfaces between adjacent printed layers. Consequently, damage is initiated earlier than in the 0° configuration, where the deposited material is more favorably aligned with the tensile loading direction, resulting in a reduced deformation capacity prior to failure.

*RGD 720* specimens were also subjected to static tensile testing at a 90° build orientation. The plot in Figure 2c shows the load–displacement relationship for ten specimens (*n* = 10) in this configuration. The specimens exhibit a more linear increase in load with increasing displacement, without a distinct peak as observed for the 0° configuration. Instead, the load increases steadily, reaching maximum values of approximately 400–500 N at displacements of around 2–3 mm. As in the other configurations, the results are relatively consistent across specimens, with minor variations in maximum load values.

These results indicate that the mechanical properties of *RGD 720* are strongly dependent on build orientation, which is critical for optimizing 3D printing processes in terms of mechanical performance. The consistency of the results across all configurations confirms good repeatability of both the manufacturing process and the experimental procedure. The specimens manufactured at the 0° build orientation exhibited a gradual failure process along the layers arranged parallel to the tensile loading direction. In contrast, specimens printed at the 45° and 90° orientations showed earlier separation between adjacent printed layers during tensile loading. The observed failure patterns are consistent with the influence of build orientation on the mechanical response of additively manufactured polymer materials; however, the underlying failure mechanisms were inferred from macroscopic observations and were not verified by microscopic analyses.

Table 2 presents the results for three different build orientations: 0°, 45°, and 90°. The data indicate that build orientation has a significant influence on the mechanical response of the material. Specimens printed at 0° and 45° exhibit higher load-carrying capacity and more favorable tensile properties, while specimens printed at 90° show a noticeable reduction in strength. Differences in elongation behavior between configurations further confirm the anisotropic nature of the material, highlighting the strong dependence of mechanical performance on build orientation. T_F_0°, T_F_45°, and T_F_90° denote tensile specimens manufactured at build orientations of 0°, 45°, and 90°, respectively. The symbol ΔL represents the maximum crosshead displacement recorded at specimen failure during the tensile test.

### 3.2. Determination of Anisotropy

According to the adopted formulations, the appropriate anisotropy coefficients were determined using Equations (4)–(11) for the first-order anisotropy coefficient and Equations (12)–(19) for the second-order anisotropy coefficient. The calculated values were arranged in matrices (10) and (18), on the basis of which the corresponding matrix determinants (11) and (19) were obtained.

To determine the first-order anisotropy coefficient (*A_I_*) Equations (4)–(11) were applied. The results demonstrated that the build orientation significantly affected the tensile behavior of the *RGD 720* material. Specimens manufactured at the 0° orientation exhibited higher load-displacement capacity and a more gradual failure process, whereas specimens printed at 45° and 90° exhibited earlier separation between adjacent printed layers during tensile loading, resulting in lower mechanical performance. The highest strength was observed at 0°, suggesting that the material performs best along the build direction. The 90° orientation exhibits the weakest mechanical properties, which may lead to unpredictable failure under loading in this direction. High anisotropy ratios, such as AI_(0°/90°)_ and *A_I_*_(45°/90°)_, indicate that the material is significantly stronger in directions parallel to the build orientation compared to perpendicular directions.

The calculated determinant values $\left|W_{AI}\right|$ and $\left|W_{AII}\right|$ should be interpreted as global descriptors of the anisotropic response rather than indicators of the mechanical performance of an individual build orientation. These parameters quantify the overall differentiation of the mechanical behavior among all investigated printing orientations by considering the complete anisotropy matrices. Therefore, they cannot be used independently to identify the optimum build orientation. Instead, the selection of the 0° configuration for the subsequent investigation was based directly on the experimental tensile results, including the highest maximum load, the largest displacement at failure, and the greatest energy absorption capacity (Table 3).

### 3.3. Tensile Behavior of Cellular Structures

**Figure 3:**
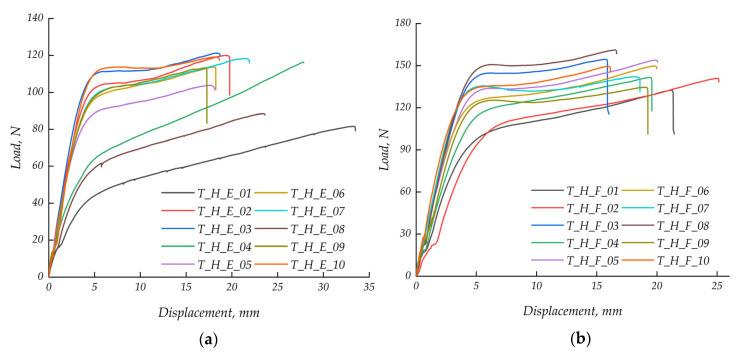
Load–displacement curves obtained during the static tensile test of hexagonal cellular structures manufactured from *RGD 720*: (**a**) specimens made of the base material; (**b**) specimens filled with molding silicone. All curves corresponding to *n* = 10 specimens are presented.

Figure 3a presents load–displacement curves for unfilled specimens with a hexagonal structure. A clear variability in the mechanical response is observed. In most cases, an initial linear increase in load occurs, corresponding to the elastic region, followed by a maximum value and a subsequent decrease in load. The character of this decrease varies from abrupt, indicating brittle failure, to more gradual, suggesting increased ductility of the structures. Differences in maximum load values and corresponding displacements are also evident, indicating non-uniform mechanical behavior of the tested specimens.

Figure 3b presents analogous curves for hexagonal structures filled with silicone. Compared to unfilled specimens, higher maximum load values and more extended curve profiles are observed. In many cases, the post-peak load reduction is more gradual, indicating an increased ability for plastic deformation and energy absorption. Thus, silicone filling improves load-bearing capacity and alters the failure mechanism from brittle to more ductile behavior.

**Figure 4:**
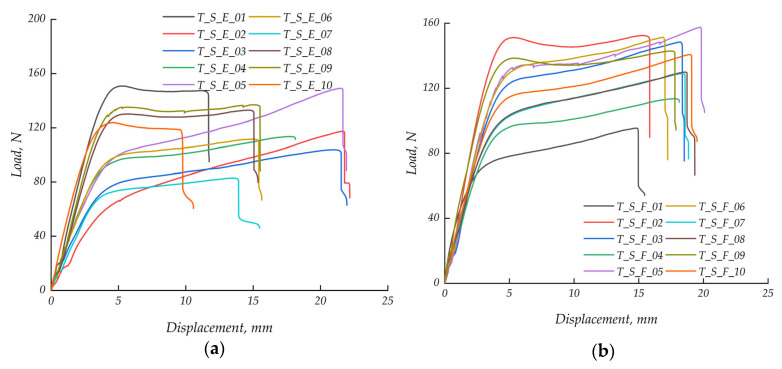
Load–displacement curves obtained during the static tensile test of spiral cellular structures manufactured from *RGD 720*: (**a**) specimens made of the base material; (**b**) specimens filled with molding silicone. All curves corresponding to *n* = 10 specimens are presented.

Figure 4a presents load–displacement curves for unfilled specimens with a spiral structure. All characteristics exhibit an initial linear region corresponding to elastic behavior, followed by a maximum load and a sudden drop. This response indicates brittle failure of the structures. Variations in maximum load and displacement values suggest variability in mechanical response, likely resulting from manufacturing inconsistencies or minor structural defects.

Figure 4b presents analogous curves for spiral structures filled with silicone. Compared to unfilled specimens, a more stable response and a more gradual post-peak load decrease are observed. This indicates an increased energy absorption capacity and a more ductile failure mode. Silicone filling reduces brittleness and decreases result dispersion, although some variability remains.

**Figure 5:**
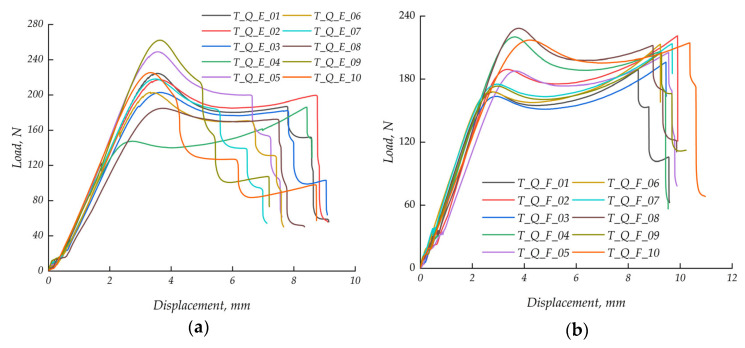
Load–displacement curves obtained during the static tensile test of quasi-self-similar cellular structures manufactured from RGD 720: (**a**) specimens made of the base material; (**b**) specimens filled with molding silicone. All curves corresponding to *n* = 10 specimens are presented.

Figure 5a presents load–displacement curves for unfilled specimens with a quasi-self-similar structure. All characteristics exhibit an initial linear increase in load corresponding to the elastic region, followed by a maximum value and a subsequent decrease. The nature of this decrease varies—from abrupt, indicating brittle failure, to more gradual, suggesting a partial ability to sustain load after damage initiation. In many cases, the material continues to carry limited loads after reaching the maximum load, indicating progressive rather than instantaneous failure. The observed variability in the curves reflects non-uniform mechanical behavior of the structures.

Figure 5b presents analogous curves for specimens filled with silicone. Compared to unfilled specimens, a more stable response and a more gradual post-peak load reduction are observed. The filling enhances the load-carrying capacity after damage initiation and reduces the abruptness of failure. In the final stage of deformation, the specimens maintain higher load values, indicating improved energy absorption capability.

Different displacement axis ranges were applied in individual figures to improve the readability of the experimental curves and accurately present the deformation behavior of each structure type.

In the analyzed cellular structures, the load–displacement response provided more representative information regarding deformation mechanisms and progressive failure behavior than conventional stress–strain parameters alone. This is primarily associated with the complex deformation modes and structural instability mechanisms characteristic of thin-walled additively manufactured cellular systems.

To improve the statistical reliability of the presented results, all analyzed configurations were tested using ten specimens (*n* = 10), and the obtained results were evaluated using mean values, standard deviations (*SD*), minimum values, and maximum values.

Table 4 summarizes the maximum tensile load $F_{max}$ and the corresponding elongation ΔL for six variants of cellular structures. To improve the statistical clarity of the presented results, the mean values, standard deviations (*SD*), minimum values, and maximum values for both load and displacement were included in Table 4. The results indicate significant differences in load-bearing capacity and deformation behavior depending on the structure type and the presence of filling. In general, specimens made solely of the base material exhibit lower strength and higher deformation, while silicone-filled specimens show increased load capacity and reduced elongation. The effect of silicone filling consistently improves mechanical performance and reduces the variability of results. Among the analyzed structures, quasi-self-similar configurations demonstrate the highest strength and the most stable deformation behavior, whereas hexagonal and spiral structures exhibit lower load-bearing capacity and greater variability. Overall, the results confirm that both structural geometry and internal filling play a crucial role in determining the mechanical response of cellular structures.

The improved deformation stability observed in silicone-filled structures may be associated with partial stress redistribution within the cellular architecture and reduced local instability of the thin walls during tensile loading. The presence of the silicone filling was accompanied by increased load-bearing capacity and more stable deformation behavior, suggesting that it contributed to delaying the onset of localized failure and reducing the sensitivity of the cellular structure to deformation localization.

In contrast, unfilled cellular structures were more susceptible to localized deformation and stress concentration effects, which promoted earlier instability and progressive failure of the thin-walled architecture.

## 4. Discussion

The results for solid specimens (F) clearly demonstrate a strong dependence of mechanical performance on build orientation in the *PolyJet Matrix* (*PJM*) technology. Initially, specimens were fabricated in three orientations (0°, 45°, and 90°) to identify the optimal direction in terms of mechanical performance. Based on these results, the 0° orientation was selected for further investigations involving cellular structures, as it provided the highest strength and most favorable mechanical response.

Specimens printed at 0° exhibit the highest load-carrying capacity and tensile strength, while those printed at 45° show slightly lower strength but a more consistent load–displacement response, as evidenced by the lower variability among the recorded tensile curves. In contrast, specimens printed at 90° demonstrate significantly reduced strength and a more brittle response. These findings confirm that the layer-by-layer deposition characteristic of PJM strongly influences interlayer bonding and, consequently, the mechanical performance, deformation stability, and anisotropic response of the material under tensile loading.

The analysis of anisotropy coefficients, derived from solid specimen data, further confirms the directional dependence of mechanical properties. Both first- and second-order anisotropy coefficients indicate pronounced anisotropy of the *RGD 720* material. The significantly higher strength observed in the 0° orientation compared to 90° highlights the dominant role of layer alignment relative to the loading direction. The mathematical model based on anisotropy matrices is consistent with the experimental results, confirming its suitability for describing the mechanical behavior of the material. Additionally, the obtained determinant values suggest potential instability in mechanical response under certain loading configurations, which should be considered during design.

The presence of silicone enhances energy absorption and promotes a transition from brittle to more ductile failure behavior, which is consistent with previous studies showing that organosilicon modifiers can significantly improve the toughness and impact resistance of polymer materials [27].

The results obtained for cellular structures demonstrate that both geometry and internal filling significantly influence load-bearing capacity, deformation stability, and post-peak mechanical response. The observed behavior indicates that structural topology and silicone filling jointly affect stress redistribution and progressive deformation mechanisms during tensile loading. Unfilled structures exhibit higher deformability but lower load-bearing capacity and greater variability, indicating susceptibility to brittle failure. In contrast, silicone-filled structures show improved strength, reduced result dispersion, and a more stable post-peak response.

From an application perspective, silicone-filled cellular structures may be particularly suitable for components requiring energy absorption, vibration damping, or improved damage tolerance. The ability to tailor mechanical response through both geometry and material filling provides a flexible design approach in additive manufacturing.

Among the analyzed configurations, quasi-self-similar structures exhibited high load–displacement capacity and relatively stable deformation behavior, making them the most favorable solution. Hexagonal and spiral structures show lower strength and higher deformation, particularly in the unfilled state, indicating less favorable mechanical performance. The least favorable case is associated with unfilled structures and specimens printed at 90°, where both strength and reliability are significantly reduced.

Overall, the best mechanical performance is achieved for specimens printed at 0° and for silicone-filled quasi-self-similar structures, while the weakest performance is observed for 90° orientation and unfilled cellular configurations. These findings confirm that both build orientation and structural design are critical factors in optimizing the mechanical properties of additively manufactured components.

In contrast to the referenced study conducted for *FDM* technology, where the mechanical performance of *PLA* was primarily optimized through process parameters and evaluated using *ANOVA*, the present work focuses on *PolyJet Matrix (PJM)* technology. In this study, the analysis was based on a structured matrix approach, enabling a systematic comparison of material configurations and structural variants. While both studies address anisotropy in additively manufactured materials, its origin differs significantly: in *FDM* it is mainly associated with interlayer bonding and process parameters, whereas in *PJM* it results from the combined effect of material properties and cellular structure design [4].

## 5. Conclusions

The conducted study confirmed that both build orientation and cellular geometry significantly influence the mechanical behavior of thin-walled structures manufactured using PolyJet Matrix technology. The 0° build orientation demonstrated the highest load-bearing capacity among the analyzed configurations. However, despite the superior tensile strength, this orientation also exhibited increased displacement variability associated with a more gradual post-peak deformation response. Based on macroscopic observations, the observed failure behavior was consistent with progressive separation between adjacent printed layers.

The application of silicone filling generally improved the mechanical response of the analyzed cellular structures by increasing load-bearing capability and enhancing deformation stability. The observed behavior suggests that silicone filling contributed to partial stress redistribution within the cellular architecture and reduced localized instability during tensile loading.

Differences in mechanical response were also strongly dependent on cellular geometry. Hexagonal and spiral structures exhibited improved load-bearing behavior after silicone filling, whereas quasi-self-similar structures demonstrated more complex deformation characteristics despite maintaining relatively high mechanical performance.

It should be emphasized that the primary objective of the present study was not to maximize the absolute tensile properties of cellular structures, but to quantitatively evaluate the influence of introducing cellular perforations on the mechanical response and to determine the extent to which silicone filling can compensate for the reduction in load-bearing capacity and deformation performance caused by the cellular architecture.

Furthermore, the proposed matrix-based anisotropy approach enabled a more comprehensive assessment of mechanical behavior by simultaneously considering force- and displacement-related parameters. The obtained results demonstrate that the influence of build orientation in photopolymer jetting technologies is more complex than may be inferred solely from conventional anisotropy coefficients.

Overall, the presented methodology and experimental observations confirm the potential of hybrid cellular structures manufactured using *PolyJet Matrix* technology for lightweight engineering applications requiring tailored mechanical properties and controlled deformation behavior.

## Figures and Tables

**Table 1:** Selected material properties according to manufacturer data [24,25].

Properties	Unit	RGD 720	Molding Silicone
Elastic modulus	$\frac{N}{{mm}^{2}}$	2000–3000	0.1–10
Poisson’s ratio	-	0.35	0.47–0.5
Shear stress	$\frac{N}{{mm}^{2}}$	30–50	0.2–0.6
Density	$\frac{kg}{m^{3}}$	1170–1190	1100–1200
Tensile strength	$\frac{N}{{mm}^{2}}$	50–65	2–5
Compressive strength	$\frac{N}{{mm}^{2}}$	60–75	3–8
Yield strength	$\frac{N}{{mm}^{2}}$	40–55	-

**Table 2:** Determined characteristics of the RGD 720 material.

	*T_F_0°*		*T_F_45°*		*T_F_90°*	
	∆*L*, mm	*Fmax*, N	∆*L*, mm	*Fmax*, N	∆*L*, mm	*Fmax*, N
$\bar{X}$	25.35	961.92	5.30	977.59	1.97	395.08
*SD*	5.06	74.96	0.14	19.72	0.27	36.95
*Max*	29.8	1153.19	5.44	1004.09	2.46	461.01
*Min*	17.47	878.94	5.00	941.83	1.65	336.89
	*So*, mm2	*Rm*, MPa	*So*, mm2	*Rm*, MPa	*So*, mm2	*Rm*, MPa
$\bar{X}$	20.12	47.80	20.08	48.79	20.08	19.67
*SD*	0.05	3.80	0.10	1.11	0.07	1.82
*Max*	20.23	57.54	20.27	50.18	20.22	22.9
*Min*	20.04	43.64	19.95	46.46	20.00	16.84

**Table 3:** Determined values of first- and second-order anisotropy.

First-Order Anisotropy		Second-Order Anisotropy	
${A_{I}}_{0{}^{\circ}/45{}^{\circ}}^{RGD\;720}$	0.98	${A_{II}}_{0{}^{\circ}/45{}^{\circ}}^{RGD\;720}$	4.71
${A_{I}}_{0{}^{\circ}/90{}^{\circ}}^{RGD\;720}$	2.43	${A_{II}}_{0{}^{\circ}/90{}^{\circ}}^{RGD\;720}$	31.33
${A_{I}}_{45{}^{\circ}/0{}^{\circ}}^{RGD\;720}$	1.02	${A_{II}}_{45{}^{\circ}/0{}^{\circ}}^{RGD\;720}$	0.21
${A_{I}}_{45{}^{\circ}/90{}^{\circ}}^{RGD\;720}$	2.47	${A_{II}}_{45{}^{\circ}/90{}^{\circ}}^{RGD\;720}$	6.66
${A_{I}}_{90{}^{\circ}/0{}^{\circ}}^{RGD\;720}$	0.41	${A_{II}}_{90{}^{\circ}/0{}^{\circ}}^{RGD\;720}$	0.03
${A_{I}}_{90{}^{\circ}/45{}^{\circ}}^{RGD\;720}$	0.40	${A_{II}}_{90{}^{\circ}/45{}^{\circ}}^{RGD\;720}$	0.15
$M_{A_{I}}$	$\left[\begin{array}{ccc} 1 & 1.02 & 0.41\\ 0.98 & 1 & 0.40\\ 2.43 & 2.47 & 1 \end{array}\right]$	$M_{A_{II}}$	$\left[\begin{array}{ccc} 1 & 1.21 & 0.03\\ 4.71 & 1 & 0.15\\ 31.33 & 6.66 & 1 \end{array}\right]$
$\left|W_{A_{I}}\right|$	0.000014	$\left|W_{A_{II}}\right|$	0.010547

**Table 4:** Summary of estimated characteristics for cellular structures.

	*T_H_E*		*T_H_F*		*T_S_E*		*T_S_F*		*T_Q_E*		*T_Q_F*	
	*F_max_*, N	∆*L*, mm	*F_max_*, N	∆*L*, mm	*F_max_*, N	∆*L*, mm	*F_max_*, N	∆*L*, mm	*F_max_*, N	∆*L*, mm	*F_max_*, N	∆L, mm
$\bar{X}$	109.60	23.25	146.06	19.81	122.30	16.97	136.13	17.68	217.4	8.30	210.92	9.70
*SD*	13.96	6.26	9.22	2.58	21.01	3.96	19.51	1.64	24.86	0.83	12.08	0.28
*Max*	121.28	33.49	161.18	25.14	150.77	22.17	157.55	20.08	262	9.14	228	10.23
*Min*	81.58	17.27	132.47	16.02	82.89	11.74	95.46	15.46	185	7.12	189	9.24

## Data Availability

The original contributions presented in this study are included in the article. Further inquiries can be directed to the corresponding author.

## Abbreviations

The following abbreviations are used in this manuscript:

PJM	PolyJet Matrix
FDM	Fused Deposition Modeling
FFF	Fused Filament Fabrication
RGD 720	Name of resin material
